# Comparison between objective measures of smoking and self-reported smoking status in patients with asthma or COPD: are our patients telling us the truth?*

**DOI:** 10.1590/S1806-37132015000004526

**Published:** 2015

**Authors:** Rafael Stelmach, Frederico Leon Arrabal Fernandes, Regina Maria Carvalho-Pinto, Rodrigo Abensur Athanazio, Samia Zahi Rached, Gustavo Faibischew Prado, Alberto Cukier

**Affiliations:** University of São Paulo, School of Medicine, Hospital das Clínicas, São Paulo, Brazil. Department of Pulmonology, Instituto do Coração - InCor, Heart Institute - University of São Paulo School of Medicine Hospital das Clínicas, São Paulo, Brazil; University of São Paulo, School of Medicine, Hospital das Clínicas, São Paulo, Brazil. Department of Pulmonology, Instituto do Coração - InCor, Heart Institute - University of São Paulo School of Medicine Hospital das Clínicas, São Paulo, Brazil; University of São Paulo, School of Medicine, Hospital das Clínicas, São Paulo, Brazil. Department of Pulmonology, Instituto do Coração - InCor, Heart Institute - University of São Paulo School of Medicine Hospital das Clínicas, São Paulo, Brazil; University of São Paulo, School of Medicine, Hospital das Clínicas, São Paulo, Brazil. Department of Pulmonology, Instituto do Coração - InCor, Heart Institute - University of São Paulo School of Medicine Hospital das Clínicas, São Paulo, Brazil; University of São Paulo, School of Medicine, Hospital das Clínicas, São Paulo, Brazil. Department of Pulmonology, Instituto do Coração - InCor, Heart Institute - University of São Paulo School of Medicine Hospital das Clínicas, São Paulo, Brazil; University of São Paulo, School of Medicine, Hospital das Clínicas, São Paulo, Brazil. Department of Pulmonology, Instituto do Coração - InCor, Heart Institute - University of São Paulo School of Medicine Hospital das Clínicas, São Paulo, Brazil; University of São Paulo, School of Medicine, Hospital das Clínicas, São Paulo, Brazil. Department of Pulmonology, Instituto do Coração - InCor, Heart Institute - University of São Paulo School of Medicine Hospital das Clínicas, São Paulo, Brazil

**Keywords:** Asthma, Pulmonary disease, chronic obstructive, Cotinine, Carbon monoxide, Smoking

## Abstract

**OBJECTIVE:**

Smoking prevalence is frequently estimated on the basis of self-reported smoking status. That can lead to an underestimation of smoking rates. The aim of this study was to evaluate the difference between self-reported smoking status and that determined through the use of objective measures of smoking at a pulmonary outpatient clinic.

**METHODS:**

This was a cross-sectional study involving 144 individuals: 51 asthma patients, 53 COPD patients, 20 current smokers, and 20 never-smokers. Smoking status was determined on the basis of self-reports obtained in interviews, as well as through tests of exhaled carbon monoxide (eCO) and urinary cotinine.

**RESULTS:**

All of the asthma patients and COPD patients declared they were not current smokers. In the COPD and asthma patients, the median urinary cotinine concentration was 167 ng/mL (range, 2-5,348 ng/mL) and 47 ng/mL (range, 5-2,735 ng/mL), respectively (p < 0.0001), whereas the median eCO level was 8 ppm (range, 0-31 ppm) and 5 ppm (range, 2-45 ppm), respectively (p < 0.05). In 40 (38%) of the patients with asthma or COPD (n = 104), there was disagreement between the self-reported smoking status and that determined on the basis of the urinary cotinine concentration, a concentration > 200 ng/mL being considered indicative of current smoking. In 48 (46%) of those 104 patients, the self-reported non-smoking status was refuted by an eCO level > 6 ppm, which is also considered indicative of current smoking. In 30 (29%) of the patients with asthma or COPD, the urinary cotinine concentration and the eCO level both belied the patient claims of not being current smokers.

**CONCLUSIONS:**

Our findings suggest that high proportions of smoking pulmonary patients with lung disease falsely declare themselves to be nonsmokers. The accurate classification of smoking status is pivotal to the treatment of lung diseases. Objective measures of smoking could be helpful in improving clinical management and counseling.

## Introduction

Cigarette smoking, the main risk factor for COPD,^(^ 1 ^)^ can aggravate the inflammation associated with asthma, causing the symptoms to be more severe, accelerating the decline in pulmonary function, and impairing the short-term therapeutic response to corticosteroids.^(^ 2 ^)^ Although self-reports of smoking status are widely used in order to estimate the prevalence of smoking in patients with asthma or COPD,^(^ 3 ^-^ 5 ^)^ their use has been shown to underestimate smoking rates, especially because of the decreasing social acceptability of smoking.^(^ 6 ^)^ Some authors have questioned the validity of self-reported smoking status in the general population and have reported significant rates of misclassification.^(^ 7 ^)^

In the city of São Paulo, Brazil, the prevalence of smoking in the adult population was reported to be 20.9% in 2008.^(^ 8 ^)^ The assessment of smoking status is pivotal to the treatment of respiratory diseases. Smoking cessation is not only regarded as the most efficient intervention to slow the progression of COPD^(^ 9 ^)^ but can also improve the management of and treatment response in patients with asthma.^(^ 2 ^)^

In smoking-cessation intervention studies, the use of a biochemical measure has been deemed essential, in order to validate self-reported smoking status.^(^ 10 ^)^ Determining the exhaled carbon monoxide (eCO) level is a rapid, noninvasive method of assessing smoking status. Although CO has a half-life of approximately 4 h and can be detectable in the blood for up to 24 h, the contribution of environmental sources cannot be distinguished from that of cigarette smoking, potentially leading to false-positive results.^(^ 11 ^)^ Several studies have shown that cut-off values between 6 and 8 ppm are appropriate to separate smokers from nonsmokers.^(^ 12 ^)^ If an individual smokes only a few cigarettes per day or has not smoked a cigarette for several hours, eCO testing can yield false-negative results.^(^ 13 ^)^ The major metabolite of nicotine is cotinine, and urinary cotinine is a specific marker for nicotine. Except in users of nicotine replacement therapy, elevated cotinine concentrations indicate tobacco use or exposure to environmental tobacco smoke.^(^ 14 ^)^ Cotinine concentrations are less dependent on the time elapsed since the last cigarette smoked than are eCO levels, because the half-life of cotinine in urine is approximately 16 h.^(^ 15 ^)^ However, the urinary cotinine concentration is highly dependent on the assay method and on the laboratory performing the analysis, making it difficult to identify a universal cut-off concentration for classifying an individual as a smoker or nonsmoker.^(^ 16 ^)^

The purpose of this study was to draw comparisons between self-reports of smoking status and the results of objective measures of smoking (urinary cotinine assays and eCO testing) in patients with stable asthma or COPD.

## Methods

### Study design 

This was a cross-sectional study involving asthma patients and COPD patients recruited from among those under regular treatment at the Pulmonary Outpatient Clinic of the Heart Institute at the University of São Paulo School of Medicine *Hospital das Clínicas*, in the city of São Paulo, Brazil. Information about smoking habits, symptoms, lifestyle, exposure, and medication usage were collected by an interviewer. All interviewers were trained to avoid pressuring or judging the patients. All subjects were assured that the results were confidential, in order to encourage accurate reporting of smoking habits. The Research Ethics Committee of the *Hospital das Clínicas* approved the study protocol, and all participants gave written informed consent.

### Subjects

The diagnoses of COPD and asthma were based on the definitions provided in the guidelines established by the Global Initiative for Chronic Obstructive Lung Disease^(^ 1 ^)^ and the Global Initiative for Asthma,^(^ 17 ^)^ respectively. Patients with asthma or COPD were recruited in person by members of the research team or interviewers after regular visits to the outpatient clinic. Inclusion criteria were having been in outpatient treatment for at least 12 months at recruitment and having had no changes in treatment regimen within the last 4 weeks. Patients using nicotine replacement therapy were excluded, as were those with any cognitive disorder that would have impaired their ability to complete a questionnaire, those with renal failure requiring dialysis, and those with facial deformities that would have impeded the use of spirometry or measurement of the eCO level. To ensure that the urinary cotinine and eCO results were reliable, we also recruited normal subjects without asthma, COPD, or other identifiable respiratory problems: 20 current smokers (positive control group) and 20 never-smokers (negative control group). The control subjects were recruited from among university students and employees, through the use of posters displayed in the hospital and university. We employed the following definitions of smoking status: a current smoker was defined as a subject who reported current, regular use of cigarettes; a never-smoker was defined as a subject who reported never having smoked cigarettes; and a former smoker was defined as a subject who reported a lifetime smoking history of ≥ 100 cigarettes and smoking abstinence for at least the last 12 months before inclusion in the study.

### Determination of self-reported smoking status

Immediately after recruitment, we conducted face-to-face interviews to collect data related to health history and demographic characteristics. Participants were asked "Do you smoke?"; "Are you smoking now?"; "When did you quit?"; "How many cigarettes do you smoke per day?"; and "How many smokers live in your household?" Responses to these questions were recorded on a flowchart as either nominal (yes/no) or interval data.

### Pulmonary function tests

For all subjects, we determined FEV_1_ and FVC using a spirometer (KoKo; nSpire Health Inc., Longmount, CO, USA). All spirometry procedures were performed in accordance with the recommendations made jointly by the American Thoracic Society and European Respiratory Society. ^(^ 18 ^)^ All pulmonary function tests were performed between 8:00 and 12:00 a.m.

### Determination of urinary cotinine concentration

To determine urinary cotinine concentrations, morning urine samples were collected from patients at the time of an appointment at the outpatient clinic. Urine samples were collected in sterile bottles. Aliquots of those samples were stored at −80°C for later batched laboratory analysis.

The quantitative analysis of cotinine in urine samples was performed with a modified HPLC method. A cotinine concentration > 200 ng/mL is considered indicative of active use of nicotine-containing products.^(^ 19 ^,^ 20 ^)^

### Determination of eCO level

The levels of eCO were measured in an exhaled breath sample with a CO tester (Micro CO; Micro Medical Ltd., Rochester, UK). The subjects were given a detailed explanation of the breath analysis test and were given the opportunity to practice. Although the test has good reproducibility,^(^ 21 ^)^ it was performed in duplicate to ensure consistency. The eCO values are expressed in ppm, 0-6 ppm indicating no smoking and > 6 ppm being suggestive of smoking.^(^ 13 ^,^ 22 ^)^ Before each test, we recorded ambient levels of CO, using the CO tester calibrated against room air with a calibration syringe. The eCO tests were performed between 8:00 and 12:00 a.m.

### Sample size calculation

The sample size was calculated with the aim of selecting a sample that would be sufficient to detect a 10% difference between self-reported smoking status and that detected by objective measurement. We thus determined that a sample of approximately 140 subjects was needed in order to achieve a power of 80% with a two-tailed significance of 0.05.

### Statistical analysis

Continuous variables are presented as mean ± standard error; nonparametric data are presented as median (interquartile range); and categorical variables are presented as absolute and relative frequencies. Chi-square tests were used in order to evaluate any discordance between self-reported smoking status and that determined through objective measures. To compare patient characteristics by smoking status, eCO level, and urinary cotinine concentration, we used Student's t-tests, the Mann-Whitney test, and one-way ANOVA. To assess the strength of associations between continuous variables related to patient characteristics, we calculated Pearson's correlation coefficient. Finally, we used stepwise logistic regression analysis to compare misclassified patients with patients who provided reliable information about their smoking status, with the objective of identifying predictors of such misclassification. The characteristics included in those analyses were age, level of education, and pulmonary function. The minimum level of significance adopted was 0.05. All statistical analyses were performed with SigmaStat software, version 3.5 (Systat Software Inc., San Jose, CA, USA).

## Results

**Figure 1 -:**
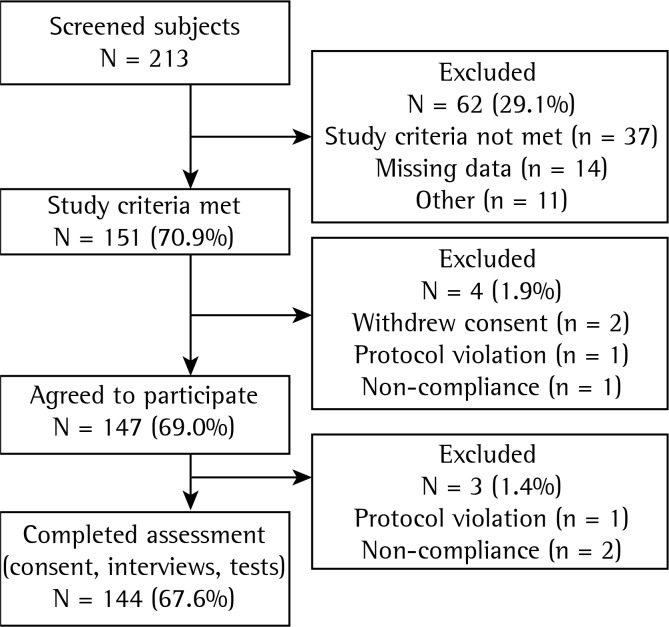
Flowchart of the sample selection process.

The recruitment flowchart is presented in Figure 1. Of the 213 eligible subjects, 69 were excluded from the analysis (for not meeting the study criteria, for not providing consent, for not complying with the protocol, or for other reasons). Therefore, the final study sample comprised a total of 144 participants (70 men and 74 women): 53 COPD patients (37 men and 16 women); 51 asthma patients (16 men and 35 women); 20 current smokers (9 men and 11 women); and 20 never-smokers (8 men and 12 women). None of the subjects enrolled in the study were using nicotine replacement therapy during the evaluation. All of the asthma patients and COPD patients declared they were not current smokers. There were 51 COPD patients and 12 asthma patients who described themselves as former smokers, stating that they had quit the habit 1-17 years prior. There were 28 COPD patients and 15 asthma patients who reported that they shared a household with one or more smokers (median, one smoker in each of the two groups). Asthma patients and COPD patients both presented with impaired pulmonary function, the mean FEV_1_ being 57% and 36% of the predicted value, respectively. This indicates that the patient portion of our study sample was composed of patients with the severe forms of their respective conditions. Clinical and functional data are presented in Table 1.

**Table 1 -:** Clinical and functional characteristics of COPD patients, asthma patients, smokers, and never-smokers.

Variable	Patients		Controls	
	COPD	Asthma	Smokers	Never-Smokers
	n = 53	n = 51	n = 20	n = 20
Gender				
Male, n (%)	37 (69.8)	16 (31.4)	9 (45.0)	8 (40.0)
Female, n (%)	16 (30.2)	35 (68.6)	11 (55.0)	12 (60.0)
Age (years), mean ± SE	64 ± 1.5*^,†,‡^	43 ± 2.0	45 ± 4.4	32 ± 3.2
FVC (% predicted), mean ± SE	86 ± 2.8	87 ± 2.6	81 ± 4.0	88 ± 2.2
FEV_1_ (% predicted), mean ± SE	36 ± 2.0*^,†,‡^	57 ± 3.2^‡^	73 ± 3.5	84 ± 1.1
FEV_1_/FVC (% predicted), median (IQR)	73 (24-92)^†,‡^	75 (24-95)^†,‡^	84 (79-88)	84 (81-87)
eCO (ppm), median (IQR)	8.0 (0-31)^†, ‡^	5.0 (2-45)^†,‡^	18 (10-45)^‡^	3.0 (1-4)
Urinary cotinine (ng/mL), median (IQR)	167 (2-5,348)*^,†,‡^	47 (5-2,735)^†,‡^	2,036 (459-3,736)*^,‡^	70 (19-179)

eCO: exhaled carbon monoxide; and IQR: interquartile range. ANOVA or Kruskal-Wallis test:

* p < 0.05 vs. asthma patients;

† p < 0.05 vs. smokers;

‡ p < 0.05 vs. never-smokers.

**Figure 2 -:**
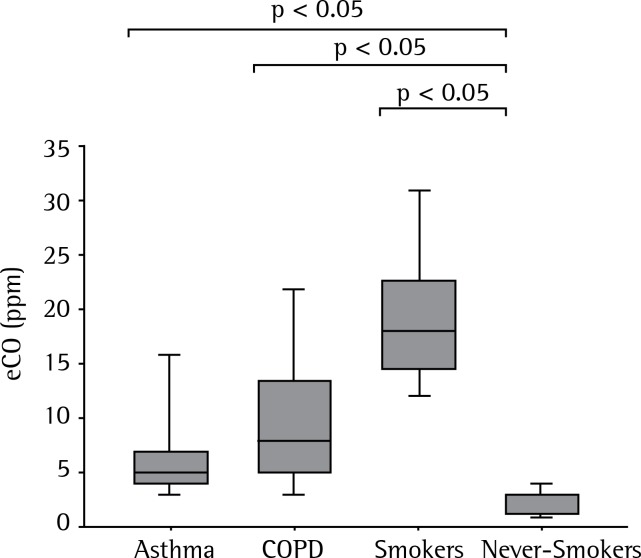
Medians and confidence intervals for exhaled carbon monoxide (eCO) in asthma patients, COPD patients, smokers, and never-smokers.

As can be seen in Figure 2, the median eCO levels of the never-smokers and current smokers were 3.0 ppm (range, 1-4 ppm) and 18 ppm (range, 10-45 ppm), respectively (p < 0.05), whereas they were 8.0 ppm (range, 0-31 ppm) and 5.0 ppm (range, 2-45 ppm), respectively, for the COPD patients and asthma patients (p < 0.05). Ambient air concentrations of CO were at 0-2 ppm during the measurements.

**Figure 3 -:**
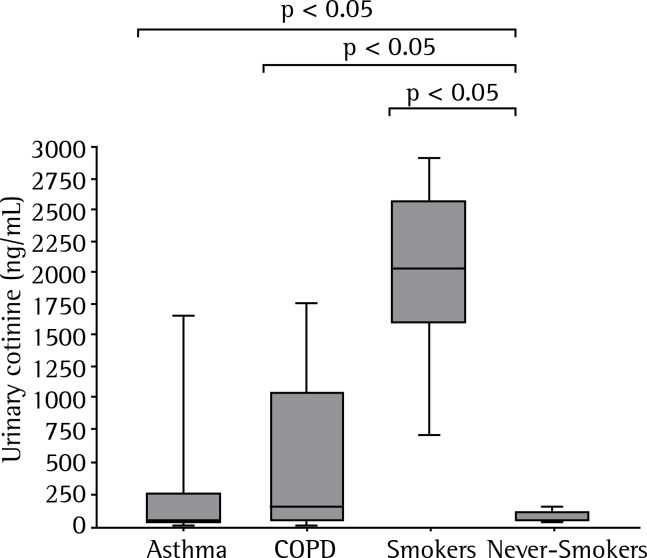
Medians and confidence intervals for urinary cotinine in asthma patients, COPD patients, smokers, and never-smokers.

Figure 3 shows the urinary cotinine concentrations. The median urinary cotinine concentration was 70 ng/mL (range, 19-179 ng/mL) in the never-smokers and 2,036 ng/mL (range, 459-3,736 ng/mL) in the current smokers, respectively (p < 0.05). In the COPD patients, the median urinary cotinine concentration was 167 ng/mL (range, 2-5,348 ng/mL), compared with 47 ng/mL (range, 5-2,735 ng/mL) for the asthma patients (p < 0.05).

All 20 of the current smokers in the positive control group tested positive for smoking, showing urinary cotinine concentrations > 200 ng/mL and eCO levels > 6 ppm. Conversely, all 20 of the never-smokers in the negative control group tested negative for smoking, by both methods.

Urinary cotinine concentrations were > 200 ng/mL in 15 asthma patients (29%) and 25 COPD patients (47%). In addition, eCO levels > 6 ppm were recorded for 16 asthma patients (31%) and 32 COPD patients (60%). Therefore, the results of the urinary cotinine assays and eCO tests, respectively, suggested that 40 (38%) and 48 (46%) of the 104 patients were misclassified as nonsmokers on the basis of their self-reports. The combination of an eCO level > 6 ppm and a urinary cotinine concentration > 200 ng/mL was identified in 7 asthma patients (14%) and 23 COPD patients (43%), collectively corresponding to 29% of the patient portion of the sample.

**Figure 4 -:**
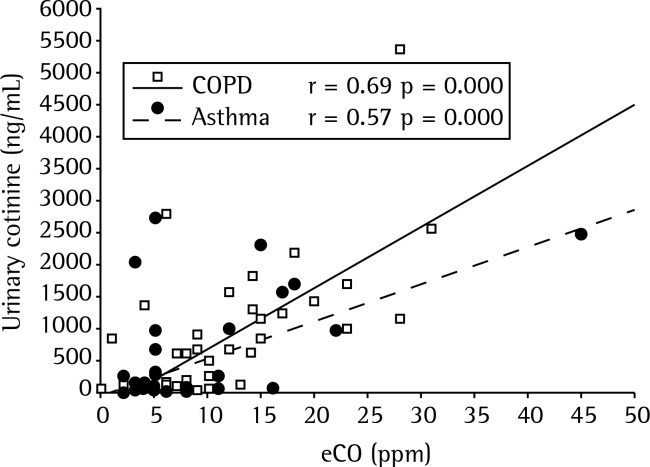
Exhaled carbon monoxide (eCO) plotted against urinary cotinine in asthma patients and COPD patients.

**Figure 5 -:**
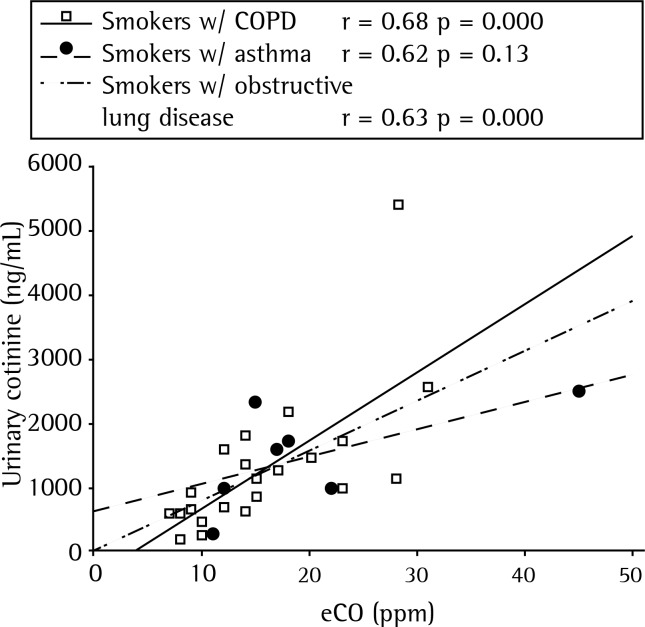
Correlation between exhaled carbon monoxide (eCO) > 6 ppm and urinary cotinine > 200 ng/mL in COPD patients (smokers w/ COPD), asthma patients (smokers w/ asthma), and both (smokers w/ obstructive lung disease).

As can be seen in Figure 4, the univariate analysis showed that eCO level correlated with urinary cotinine concentration: overall (r = 0.43, p = 0.05); in asthma patients (r = 0.57, p < 0.0001); and in COPD patients (r = 0.69, p < 0.0001). When we analyzed only the patients who were identified as smokers (Figure 5), we found that an eCO level > 6 ppm correlated significantly with a urinary cotinine concentration > 200 ng/mL in the COPD patients (r = 0.68, p < 0.0003), although not in the asthma patients (r = 0.62, p = 0.13). Analyzing the identified-as-smoking asthma and COPD patients collectively (Figure 5), we found that there was still a strong correlation between an eCO level > 6 ppm and a urinary cotinine concentration > 200 ng/mL (r = 0.63, p < 0.0001). The stepwise logistic regression, adjusted for patient characteristics such as age, level of education, exposure to passive smoking, and pulmonary function, identified no predictors of smoking status misclassification.

## Discussion

The results of the present study suggest that patients with asthma or COPD commonly provide misinformation regarding their smoking status. Although that behavior was more prominent among COPD patients, asthma patients also underreported the smoking habit. We have also shown that measuring eCO identifies most smokers, and that eCO correlates significantly with urinary cotinine. It is noteworthy that the correlation between eCO and urinary cotinine was not statistically significant among the asthma patients (p = 0.13). This might be due to a lack of power (type II error), given that few asthma patients tested positive for urinary cotinine. When the COPD and asthma patients were evaluated as a group, that correlation was significant. Few previous studies exploring the association between obstructive lung diseases and smoking behavior have used the highly sensitive and specific methods of measuring urinary cotinine and eCO for biochemical validation.

Considering the critical aspect of smoking status for the clinical management of COPD and asthma, we find it surprising that there is such a paucity of studies on the invalidity of self-reported nonsmoking by "true" smokers among patients with obstructive lung diseases. In the present study, we found that, although all of the patients described themselves as nonsmokers, 38% (47% of the COPD patients and 29% of the asthma patients) showed urinary cotinine concentrations > 200 ng/mL, a value strongly associated with current smoking.^(^ 20 ^)^

A study conducted in Spain showed that 17% of all patients seen at a respiratory medicine clinic continued to smoke while denying doing so; a higher proportion (34%) was observed in the patients with COPD.^(^ 23 ^)^ In another study, conducted in France, the measurement of cotinine in patients being treated with home oxygen therapy allowed the authors to identify 43 smokers (17% of the sample as a whole) who had theretofore described themselves as nonsmokers.^(^ 24 ^)^ In contrast, a study conducted in Japan showed that, of 351 patients with COPD or asthma, only 11 (2 with asthma and 9 with COPD) claimed to be nonsmokers and had a serum cotinine level > 50 ng/mL, which is suggestive of current smoking.^(^ 25 ^)^ These results suggest that cultural differences play a role in the proportion of patients who attempt to hide their smoking habits from health care practitioners.

The inclusion of positive and negative control groups in our study was of great importance for discussing cut-off points in the population under study. In our positive (smoker) control group, the lowest urinary cotinine concentration was 458 ng/mL, and none of the subjects had an eCO level < 10 ppm. Conversely, most of the subjects in the negative (never-smoker) control group had a cotinine concentration < 100 ng/mL, and none had an eCO level > 6 ppm. False-positive eCO results were obtained in 6 COPD patients and in only 1 asthma patient. All false-positive results were within the 7-10 ppm range, which is usually observed in light smokers. All patients with false-positive results shared a household with a smoker. Therefore, these results could be explained by environmental exposure to tobacco smoke. Exposure to pollution and underlying inflammatory lung diseases are also potential reasons for false-positive eCO testing results.

In a previous survey conducted at our institution, an eCO level ≥ 6 ppm was shown to have the greatest sensitivity and specificity for differentiating between smokers and nonsmokers.^(^ 13 ^)^ However, when trying to identify misreporting of smoking status by patients with obstructive lung diseases, these clear-cut differences disappear, and there is a boundary where overlapping occurs. If we used the highest eCO cut-off points suggested in the literature (11 ppm for COPD and 10 ppm for asthma), 32% of our COPD patients and 40% of our asthma patients would be falsely classified as nonsmokers, despite having urinary cotinine concentrations > 200 ng/mL. However, a high proportion of our patients with urinary cotinine concentrations < 100 ng/mL had eCO levels between 6 ppm and 8 ppm, which underscores the difficulty in establishing an appropriate cut-off point for eCO. This suggests that the cut-off level should vary among populations, and that borderline results should be evaluated with care in clinical practice, especially because the level of environmental exposure to tobacco smoke is likely to be high among asthma patients.^(^ 26 ^)^

The accurate determination of smoking status is pivotal to the treatment of asthma and COPD. A "real-life" study on the effectiveness of smoking cessation therapy in Brazil showed that respiratory comorbidities were not associated with treatment failure.^(^ 27 ^)^ Counseling and pharmacologic treatment can change patient smoking status and improve the course of the lung disease.

The present study has certain limitations. We did not obtain patient histories regarding passive smoking outside the home, the last cigarette smoked, or smoking patterns. Those factors can influence eCO levels and could decrease the sensitivity and specificity of eCO monitoring. In addition, we did not collect demographic data related to ethnic or racial characteristics. Ethnic or racial differences in the metabolism and clearance of nicotine could constitute an alternative explanation for our findings. There have been reports that race can influence cotinine concentrations, serum cotinine levels being higher among black smokers than among white smokers, due to differences in the metabolism of nicotine.^(^ 28 ^)^ If the Brazilian population metabolizes nicotine more slowly, our results would be partly accounted for without underreporting. Another explanation is that airway obstruction might influence levels of eCO. Therefore, eCO measurements could be inaccurate in patients with severe airway obstruction.^(^ 29 ^)^ Nevertheless, although the patients in our sample had moderate-to-severe airway obstruction, we found no significant correlation between eCO and FEV_1_. Another potential limitation of our study is that we used a convenience sample of consecutive participants, rather than a random sample of subjects.

The results of our study confirm that patient-offered smoking history is unreliable, because we did not find that patient-reported smoking status correlated with urinary cotinine concentration or eCO level. Our findings also indicate that eCO is sufficient to discriminate between smokers and nonsmokers. If our findings can be generalized to other populations and diseases, then there is cause for concern about the use of questionnaires as the only sources of data on smoking in epidemiological studies and surveys involving respiratory patients.

In summary, the present study further substantiates the idea that self-reported smoking status is unreliable in the population of patients with obstructive lung diseases, given that a considerable proportion of our patients lied to their physicians. Objective measurement of smoking status could be helpful in allowing better clinical management and patient counseling in COPD and asthma.
